# A Carbamoyl Phosphate Synthetase II (CPSII) Deletion Mutant of *Toxoplasma gondii* Induces Partial Protective Immunity in Mice

**DOI:** 10.3389/fmicb.2020.616688

**Published:** 2021-01-14

**Authors:** Xunhui Zhuo, Kaige Du, Haojie Ding, Di Lou, Bin Zheng, Shaohong Lu

**Affiliations:** ^1^Department of Immunity and Biochemistry, Institute of Parasitic Disease, Hangzhou Medical College, Hangzhou, China; ^2^Department of Immunity and Biochemistry, Institute of Parasitic Disease, Zhejiang Academy of Medical Sciences, Hangzhou, China

**Keywords:** *Toxoplasma gondii*, carbamoyl phosphate synthetase II, vaccine, immunization, CRISPR/Cas9

## Abstract

*Toxoplasma gondii* is an obligate intracellular protozoan parasite. *T. gondii* primarily infection in pregnant women may result in fetal abortion, and infection in immunosuppressed population may result in toxoplasmosis. Carbamoyl phosphate synthetase II (CPSII) is a key enzyme in the *de novo* pyrimidine-biosynthesis pathway, and has a crucial role in parasite replication. We generated a mutant with complete deletion of CPSII via clustered regularly interspaced short palindromic repeats (CRISPR)/cas9 in type-1 RH strain of *T. gondii*. We tested the intracellular proliferation of this mutant and found that it showed significantly reduced replication *in vitro*, though CPSII deletion did not completely stop the parasite growth. The immune responses induced by the infection of RHΔCPSII tachyzoites in mice were evaluated. During infection in mice, the RHΔCPSII mutant displayed notable defects in replication and virulence, and significantly enhanced the survival of mice compared with survival of RH-infected mice. We tracked parasite propagation from ascitic fluid in mice infected with the RHΔCPSII mutant, and few tachyzoites were observed at early infection. We also observed that the RHΔCPSII mutant induced greater accumulation of neutrophils. The mutant induced a higher level of T-helper type-1 cytokines [interferon (IFN)-γ, interleukin (IL)-12]. The mRNA levels of signal transducer and activator of transcription cellular transcription factor 1 and IFN regulatory factor 8 were significantly higher in the RHΔCPSII mutant-infected group. Together, these data suggest that CPSII is crucial for parasite growth, and that strains lack the *de novo* pyrimidine biosynthesis pathway and salvage pathway may become a promising live attenuated vaccine to prevent infection with *T. gondii*.

## Introduction

*Toxoplasma gondii* is an intracellular parasitic protozoon that can infect a wide range of homeothermic animals including human beings (Dubey et al., 2020). Humans acquire *T. gondii* infection usually through oral intake of oocysts in vegetables or water, or ingestion of tissue cysts in raw or undercooked meat (Blume and Seeber, 2018). Toxoplasmosis is usually asymptomatic in most immunocompetent adults, but immunodeficient individuals (e.g., organ-transplant recipients and human immunodeficiency virus-infected patients) are at risk (Wang et al., 2017; Odeniran et al., 2020). Infection by *T. gondii* can cause severe consequences in pregnant women, such as miscarriage (Garnaud et al., 2020). In addition, toxoplasmosis in agricultural animals (e.g., pigs) can lead to great economic losses and cause public-health issues around the world; recent prevalence surveys indicated that infection with *T. gondii* remains relatively high in pigs (De Berardinis et al., 2017; Suijkerbuijk et al., 2019).

Prevention or cure of toxoplasmosis is difficult because the parasite lifecycle is very complex. Transmitted oocysts shed by cats are key sources for infection in humans and animals. Tissue cysts, in general, last lifelong in hosts and are important routes of transmission (Elmore et al., 2010; Hill and Dubey, 2016). Currently, toxoplasmosis is treated by pharmacologic agents such as pyrimethamine and sulfadiazine (Deng et al., 2019). However, most marketed drugs do not work in chronic infections of *T. gondii* and cause severe side effects. In addition, drug-resistant parasites have been reported recently, along with increased concerns about treatment failure and increased clinical severity in immunocompromised patients (Montazeri et al., 2018). Therefore, the “ideal” drugs and efficacious therapies for *T. gondii* infection should be discovered and developed.

Vaccination is considered to be another effective method for preventing and controlling toxoplasmosis by providing specific antibodies and inducing cytokines directly against pathogens. Scientists have worked on developing vaccines against *T. gondii* using different strategies, such as recombinant protein- and DNA-based vaccines and dead parasites (Foroutan et al., 2019; Hajissa et al., 2019; Wang et al., 2019). Mice immunized with *T. gondii* extracts or dead parasites may fail to develop robust immune response to reinfection with *T. gondii* (Butcher and Denkers, 2002; Fox and Bzik, 2015). However, Rahman et al. (2019) found that *T. gondii* lysate antigens trigger significant immune responses leading to parasite reduction in pigs upon challenge infection, even though the mechanism of parasite clearance from tissues by early immune response needs further investigation (Rahman et al., 2020). Several studies have demonstrated that subunit vaccines and DNA vaccines induce robust humoral and cellular immunity in mice and provide partial protection against infection by *T. gondii* (Gatkowska et al., 2018; Zheng et al., 2019). For example, Wu et al. (2019) found that immunization of mice with recombinant plant-like calcium-dependent protein kinase 3 could produce humoral and cellular immune responses to acute toxoplasmosis with a prolonged survival time and significantly reduced number of cysts in brain tissues. However, these vaccines do not provide sufficient anti-toxoplasmosis protection because of the parasite’s complex mechanism for escaping immune detection in hosts.

“Attenuated parasites” with genetic alterations may offer an efficient means for vaccine development. The only commercial attenuated vaccine against toxoplasmosis (ToxoVax^®^ ; Intervet, Boxmeer, Netherlands) is available for ewes, even though the high cost and side effects limit the use of this vaccine (Verma and Khanna, 2013). Recently, several studies have documented the potential protection proffered by attenuated vaccines against toxoplasmosis in mice. Wang et al. (2020) developed a mutant with a *tkl1* deletion in a *T. gondii* RH strain that elicited strong humoral and cellular immunity in mice whose brain-cyst burden was reduced significantly after infection with oocysts. Thus, the use of attenuated vaccines could be efficacious.

*Toxoplasma gondii* is an obligate intracellular protozoan that derives most of its nutrients from host cells, including pyrimidine, which is necessary for parasite replication (Hortua Triana et al., 2016; Beraki et al., 2019). *T. gondii* has a pyrimidine-salvaging pathway and *de novo* pyrimidine-biosynthesis pathway that starts with aspartate and glutamine (Fox and Bzik, 2002). Carbamoyl phosphate synthetase II (CPSII) is a predominant enzyme of the *de novo* pyrimidine-biosynthesis pathway. CPSII possesses a unique enzyme architecture that is absent in bacteria and mammals (Fox et al., 2009). CPSII has a bifunctional N-terminal glutamine amidotransferase domain fused with C-terminal carbamoyl-phosphate synthase domains, and accepts glutamine as a donor of amine groups (Fox and Bzik, 2003; Fox et al., 2009). CPSII deletion induces severe uracil auxotrophy with loss of replication and an inability to establish acute infection in mice. Therefore, a strain with knockout of CPSII expression is severely attenuated (Fox and Bzik, 2002). Mutant, with disrupted function of CPSII enzyme, can be used as a live-attenuated vaccine and is able to elicit long-term immunity to lethal acute or chronic *T. gondii* infection in mice (Gigley et al., 2009a,b, but the protection is not always 100%, especially when challenged with type II strain (Gigley et al., 2009b). Thus, we wonder if completely deleting the CPSII gene would produce a more effective attenuated vaccine. To exclude the influence of any residue of the CPSII enzyme, we generated a mutant with complete CPSII deletion in the RH strain via a clustered regularly interspaced short palindromic repeats (CRISPR)/cas9 system, and evaluated the immune protective effect of this strain in a mouse model.

## Materials and Methods

### Ethical Approval of the Study Protocol

Animals were maintained according to the Animal Ethics Procedures and administration of Affairs Concerning Experimental Animals of the People’s Republic of China. Animal experiments were approved by the Animal Care and Use Committee of Zhejiang Academy of Medical Sciences (Zhejiang, China).

### Plasmid Construction

All primers used in this study are listed in Supplementary Table 1. The CRISPR plasmid was generated by replacing the UPRT targeting guide RNA (sgRNA) in pSAG1-CAS9-sgUPRT (kindly provided by Professor Shen Bang) with the corresponding gRNAs using site-directed mutagenesis (New England Biolabs, Ipswich, MA, United States) as described by Shen et al. (2017). Briefly, two sgRNA-targeted CPSII (5′ and 3′) were inserted into the pSAG1-CAS9-sgUPRT plasmid by replacing the UPRT sgRNA. Polymerase chain reaction (PCR) amplified the U6-sgRNA region from 5′ CPSII sgRNA plasmid using the primers gRNA2-Fw-*Kpn*I and gRNA2-Rv-*Xho*I as listed in Supplementary Table 1. The PCR products were digested with *Kpn*I (New England Biolabs) and *Xho*I (New England Biolabs) and the fragment (678 bp) was purified. In parallel, the 3′ CPSII sgRNA plasmid was digested with *Kpn*I and *Xho*I, and the fragment (9663 bp) was purified. the purified PCR products were ligated into the prepared 3′ CPSII sgRNA plasmid backbone to yield the dual sgRNA plasmid. The selection marker DHFR were amplified with primers containing 40 bp of homology to CPSII gene.

### Parasite Culture, Transfection, and PCR Diagnosis

Tachyzoites of the RH strain were maintained in human foreskin fibroblasts in complete medium (CM) composed of Dulbecco modified Eagle medium (Gibco; Thermo Fisher Scientific, Inc., Waltham, MA, United States) supplemented with 5% fetal calf serum (Gibco), penicillin (100 U/mL; Life Technologies, Gaithersburg, MD, United States), and streptomycin (100 μg/mL; Life Technologies), purchased from American Tissue Type Collection (Manassas, VA, United States). Parasites were electroporated in a 4 mm gap cuvette by the manufacturer Gene Pulser Xcell (BioRad, Hercules, CA, United States) following the protocol (1700 V, 176 μs of pulse length, two pulses with100 ms interval) as described by Shen et al. (2017). CPSII-deleted parasites were selected with pyrimethamine (Selleck Chemicals, Houston, TX, United States) and cloned by limiting the dilution.

Single positive clones were identified by diagnostic PCRs and primers are listed in Supplementary Table 1. The PCRs were carried out by the conditions with an initial melting step at 98°C for 5 min, followed by 30 cycles with each cycle at 98°C for 15 s, 61°C for 30 s, and 72°C for 45 s, followed by a final extension at 72°C for 5 min.

### Virulence Testing in Mice

Twenty adult (6–8 weeks) BALB/c mice or 20 ICR mice were obtained from the Experimental Animal Center of Zhejiang Academy of Medical Sciences. Tachyzoites of the RH strain and CPSII-deleted parasites were washed in phosphate-buffered saline (PBS). Three groups of mice were injected (i.p.) with 0.1 mL of PBS containing 1 × 10^4^ tachyzoites of the RH strain or CPSII-deleted parasites. Mice survival was monitored daily, and the survival test was carried out thrice.

### Measurement of Parasite Burden

A total of 95 adult ICR mice were randomly divided into three groups: PBS group (25 mice), WT-RH group (35 mice), and ΔCPSII group (35 mice). Three groups of mice were injected (i.p.) with 0.1 mL of PBS or PBS containing 1 × 10^4^ tachyzoites of the RH strain or CPSII-deleted parasites. Mice from each group were euthanized at days 4 and 10 post-infection (dpi) via CO_2_ overdose according to the Animal Ethics Procedures. Liver tissues were collected from each mouse and DNA extracted by using the DNeasy Blood & Tissue Kit (QIAGEN, Shanghai, China) according to the manufacturer’s protocol. Amplification of parasite DNA was carried out by using primers targeted *T. gondii* repetitive 529 bp gene as listed in Supplementary Table 1. Each reaction mixture contained 12.5 μL of 2 × Real time PCR Master Mix (Toyobo, Shanghai, China), 0.5 μL of each primer (10 μM), 1 μL DNA template, and 5.5 μL of sterile distilled water and was performed on a CFX96 Touch^TM^ Real-Time PCR Detection System (BioRad). Parasite equivalents were determined through a standard curve.

In parallel, the ascitic fluid of mice was collected at 4 and 10 dpi with 5 mL of PBS. Parasites were obtained by centrifugation at 300 *g* for 5 min at room temperature. The number of tachyzoites from each mouse was calculated using a cell counter.

### Cytokine Measurement

After mice had been injected (i.p.) with 0.1 mL of PBS containing 1 × 10^4^ tachyzoites of RH or CPSII-deleted parasites, serum samples were collected from mice at 2, 4, 8, and 10 dpi for measurement of cytokine levels. Levels of interferon (IFN)-γ and interleukin (IL)-12 were detected using an enzyme-linked immunosorbent assay (ELISA) according to manufacturer (Bio-Swamp, Wuhan, China) instructions. The optical density of each well was measured at 450 nm by a microtiter plate reader (ELX 800; Bio-Tek Instruments, Waltham, MA, United States).

### White Blood Cell (WBC) Counts

Blood samples were collected from different mouse groups at 1, 4, and 10 dpi. Each 20 μL of sample was added directly to a dilution solution, and white blood cells (WBCs) were counted by an automated machine (Celltac E; Nihdon Kohden, Tokyo, Japan).

### Immunofluorescence Assay

Vero cells, maintained in DMEN (Gibco) supplemented with 2% fetal calf serum (Gibco), penicillin (100 U/mL; Life Technologies), and streptomycin (100 μg/mL; Life Technologies), were added with or absent with uracil after infection with tachyzoites of the ΔCPSII strain. Then, cells were fixed in 4% paraformaldehyde overnight at 4^*o*^C, followed by permeabilization with 0.1% Triton X-100 in PBS and blockade with 1% bovine serum albumin in PBS for 1 h at 37°C. After washing thrice with PBS, anti-Dense Granule Protein 7 (GRA7) antibodies were diluted in the same blocking buffer and incubated for 60 min at 37°C. Alexa 594-conjugated anti-rabbit secondary antibodies were added and incubated for 60 min at 37°C. Cell nuclei were stained with 4′,6-diamidino-2-phenylindole. Then, samples were examined under a confocal laser scanning microscope (IX81-FV1000; Olympus, Tokyo, Japan).

### Statistical Analyses

The Kaplan–Meier product limit test was used to measure significant differences between survival curves (GraphPad Prism software 8.0). The parasite burdens and the mRNA expression levels of different groups were subjected to Student’s *t*-test, and the percentage and the absolute number of neutrophils and lymphocytes and the production of IFN-γ and IL-12 from different groups were all analyzed with two-way ANOVA via Tukey’s multiple comparison test. In all analyses, *p* < 0.05 was considered statistically significant.

## Results

### Generation of the CPSII Knockout Strain Using the CRISPR/Cas9 System

**FIGURE 1:**
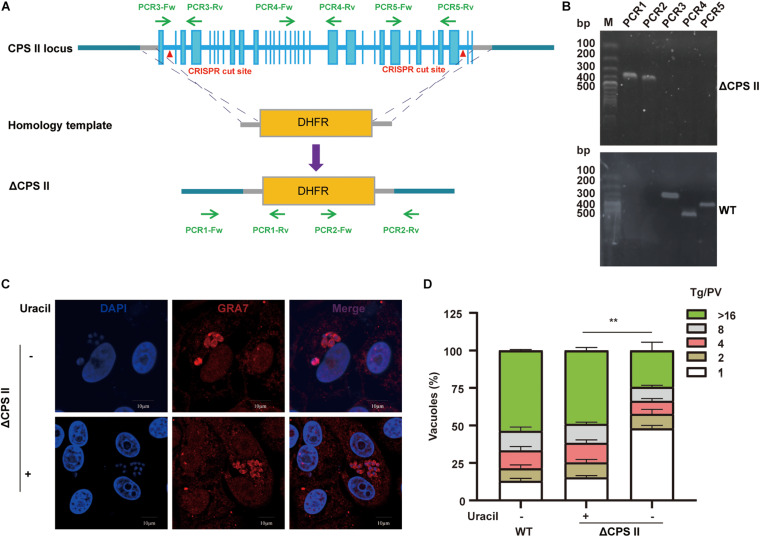
Generation and characterization of a CPSII deletion mutant. **(A)** Schematic illustration of knocking out CPSII by CRISPR/Cas9-mediated homologous gene replacement in the RH Δ*ku80* strain. **(B)** Diagnostic PCRs on a ΔCPSII clone. **(C)** Observation of intracellular replication of ΔCPSII RH strain with or without the addition of 200 μM uracil at 24 hpi by IFA using anti-GRA7 rabbit polyclonal antibody. **(D)** Intracellular replication assay of ΔCPSII RH strain with or without the addition of 200 μM uracil. The number of parasites in each parasitophorous vacuole (PV) was determined at 48 hpi. Values are shown as means ± SEM from three independent experiments. **P < 0.01 by two-way ANOVA.

The strategy for deleting the entire CPSII gene by CRISPR/CAS9-mediated homologous recombination is illustrated in Figure 1A. To avoid the effect of CPSII residues, we replaced the entire sequence of CPSII with DHFR-TS^∗^ by two sgRNAs. Plasmid templates and homology templates were transfected into the Δ*ku80* RH strain, and selected with pyrimethamine. Single clones were obtained by limiting the dilution and assessed by PCR1/2/3/4/5 to check correct replacement of the target gene by the selection marker DHFR-TS^∗^ (Figure 1A). We used three pairs of primers targeting areas in genomic CPSII far away from each other to confirm successful knockout of the entire gene. Diagnostic PCRs for one clone of RHΔCPSII are shown in Figure 1B. The PCR product of CPSII5′UTR-DHFR-CPSII3′UTR, which is performed by using CPSII-DHFR primers, was sent for sequencing for further confirmation. The results are shown in Supplementary Figure 1, and the sequences corresponded to the DHFR-TS^∗^ gene. Taken together, these results demonstrated complete deletion of the CPSII gene.

### Impact of CPSII on Tachyzoite Growth *in vitro*

Cells infected with the ΔCPSII strain underwent uracil supplementation, and tachyzoites egressed at 72 h. In cells without uracil supplementation, tachyzoites did not egress from cells at any time (Supplementary Figure 2). Interestingly, without uracil supplementation to cells, the ΔCPSII strain had a slow growth tendency because two to four tachyzoites were observed in single parasitophorous vacuole membrane (Figure 1C). Next, we undertook an intracellular replication assay to evaluate parasite proliferation *in vitro*. We found that, without the addition of uracil, growth was notably reduced (Figure 1D). These results suggested that CPSII knockout impaired the intracellular proliferation of *T. gondii*.

### Deletion of CPSII in *T. gondii* Leads to Reduced Virulence in Mice

**FIGURE 2:**
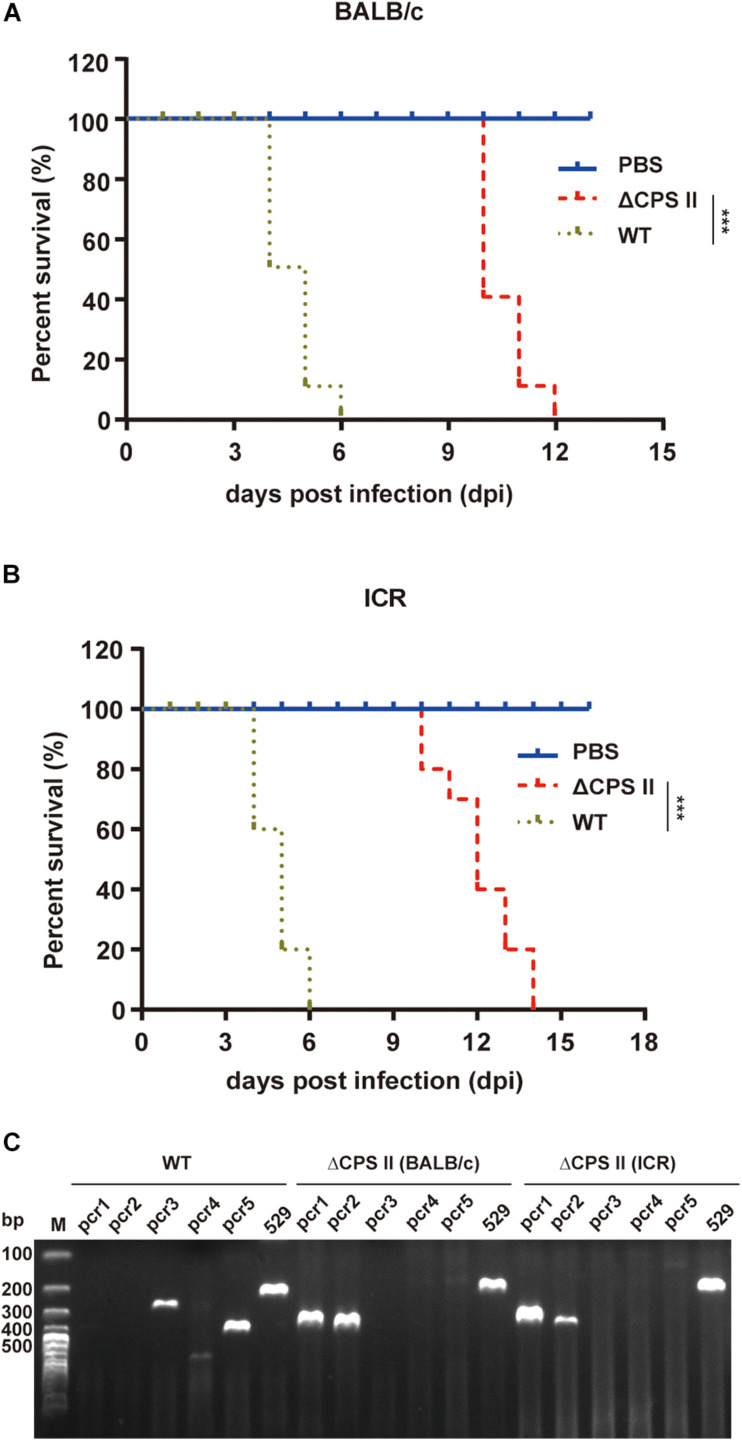
Virulence test of ΔCPSII RH strain in mice. BALB/c **(A)** or ICR **(B)** mice were infected through intraperitoneal injection with 10^4^ tachyzoites of RH strain or ΔCPSII RH strain, each group comprised 10 mice. ***p < 0.001, Kaplan–Meier product limit test. **(C)** Diagnostic PCRs of ascitic fluid samples collected from toxoplasma-infected mice.

Mice infected with the ΔCPSII strain lived longer (average, 12 days) compared with those infected with the WT RH strain (average, 5 days) (Figures 2A,B). The virulence test was carried out three times and the results were similar.

To exclude the possibility that the ΔCPSII strain was mixed with the WT RH strain, we performed PCR of tachyzoites collected from infected mice. PCR3/4/5 was negative in ΔCPSII-infected mice (Figure 2C), which suggested that RH tachyzoites were not mixed into ΔCPSII isolates. These results suggested that the virulence of the ΔCPSII strain was weakened significantly, but that the replication ability was not completely stopped.

### Parasite Burden in Mice

**FIGURE 3:**
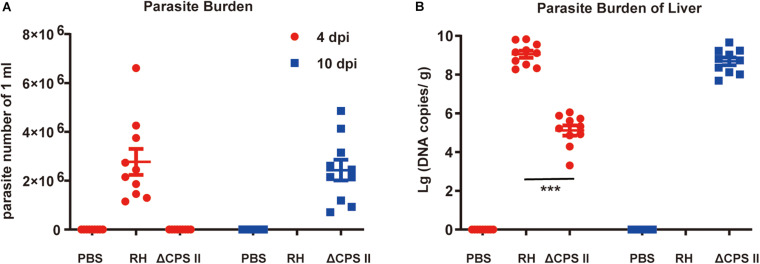
Parasite burden in ΔCPSII RH strain-infected mice. **(A)** Parasites in ascitic fluid collected from RH strain or ΔCPSII RH strain-infected ICR mice at 4 or 10 dpi were calculated by hemocytometers; each point means one mouse. **(B)** DNA copies of *T. gondii* in liver tissues 4 or 10 dpi were determined by real-time PCR based on 529 bp; each point means one mouse. ***p < 0.001 by Student’s *t*-test.

Ascitic fluid samples were collected from different mouse groups at 4 and 10 dpi, and the number of tachyzoites was calculated using a hemocytometer. The average number of parasites in WT RH strain-infected mice was 2.7 ± 0.5 × 10^6^, whereas parasites were not observed in ΔCPSII strain-infected mice at 4 dpi (Figure 3A). The average number of parasites from ΔCPSII strain-infected mice reached a similar level at 10 dpi (2.7 ± 0.5 × 10^6^) compared with that from WT RH strain-infected mice at 4 dpi. Parasite loads in spleen tissues were calculated by real-time PCR based on repetitive 529 bp gene, and we found a similar pattern to that in fluid samples (Figure 3B). Parasite loads in WT RH-infected mice were increased notably at 4 dpi whereas a similar level in ΔCPSII strain-infected mice was not observed until 10 dpi. Taken together, these results suggested that deletion of CPSII significantly reduced proliferation efficiency of *T. gondii* in mice.

### Increased Number of Neutrophils Upon Infection of the ΔCPSII Strain

**FIGURE 4:**
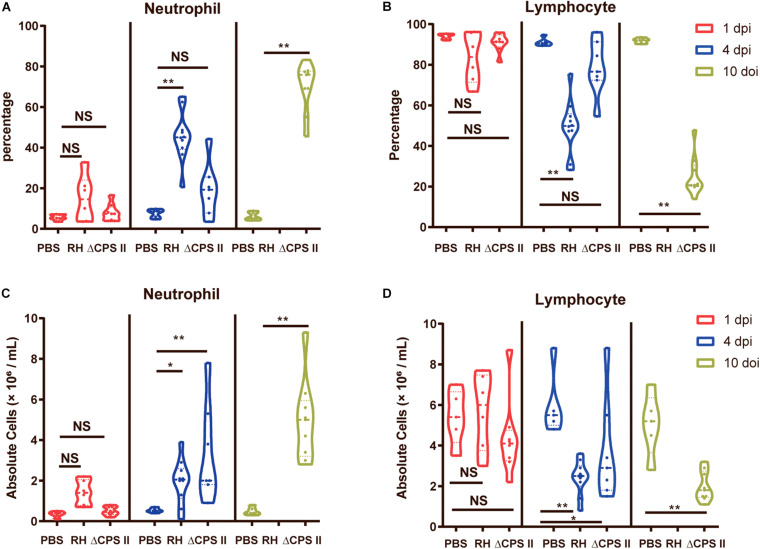
Percentage of neutrophils **(A)** and lymphocytes **(B)** and absolute number of neutrophils **(C)** and lymphocytes **(D)** in RH strain or ΔCPSII RH strain-infected ICR mice at 1, 4, or 10 dpi; each point means one mouse; NS, no significant, *p < 0.05, **p < 0.01 by two-way ANOVA.

The percentage and the absolute numbers of WBCs from blood samples were calculated with an auto-analyzer. We found a significant increase in the percentage and the absolute number of neutrophils at 10 dpi in ΔCPSII strain-infected mice, whereas the percentage and the absolute number of lymphocytes were decreased notably (Figure 4). Neutrophil accumulation induced by infection due to the ΔCPSII strain indicated partial activation of adaptive immunity, which may provide protection against toxoplasmosis.

### Increased Production of IFN-γ and IL-12 in ΔCPSII Strain-Infected Mice

**FIGURE 5:**
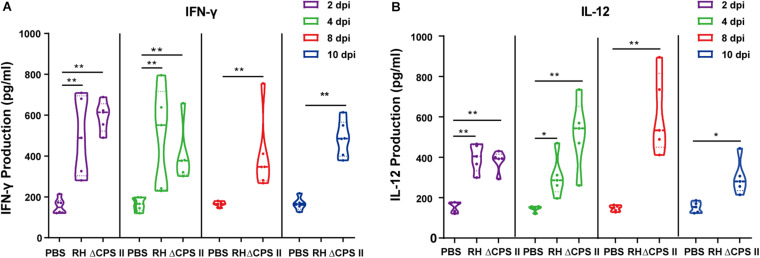
IFN-γ **(A)** and IL-12 **(B)** production in RH strain or ΔCPSII RH strain-infected ICR mice at 2, 4, 8, or 10 dpi; each point means one mouse; *p < 0.05, **p < 0.01 by two-way ANOVA.

**FIGURE 6:**
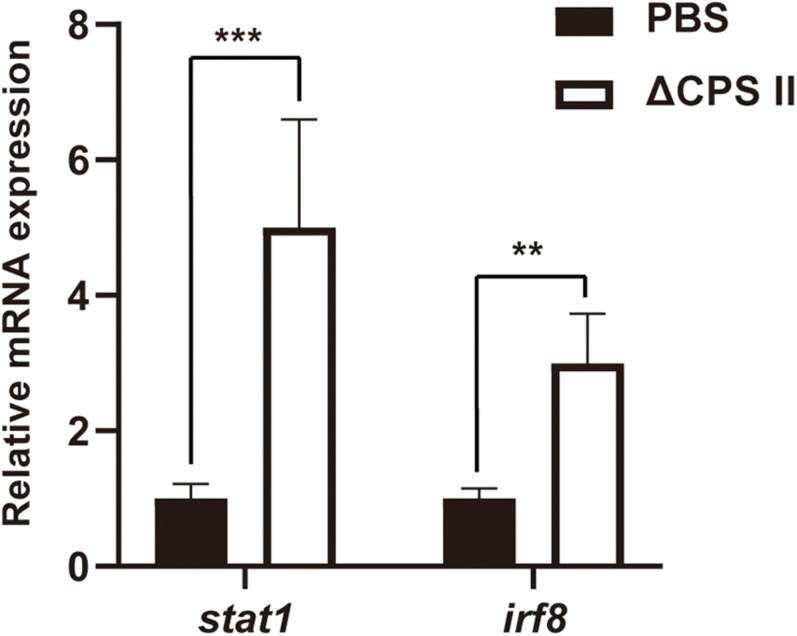
The mRNA level of *stat1* and *irf8* in PBS or ΔCPSII RH strain-infected ICR mice at 10 dpi was evaluated by real-time PCR; *n* = 5, **p < 0.01, ***p < 0.001 by Student’s *t*-test.

Production of IFN-γ and IL-12 was evaluated using ELISA kits. Production of IFN-γ and IL-12 was significantly higher in infected mice compared with that in the control group (Figure 5). Then, we measured mRNA expression of signal transducer and activator of transcription cellular transcription factor (*stat*)1 and IFN regulatory factor (*irf*)8 using real-time PCR. We found a remarkable increase in expression of *stat1* mRNA and *irf8* mRNA compared with that in the control group (Figure 6). These results suggested that *stat1* and *irf8* induced by the infection by the ΔCPSII strain were likely involved in the increased production of IFN-γ and IL-12.

## Discussion

For the last decade, numerous types of vaccines against toxoplasmosis have been evaluated in mice models. Most DNA vaccines and recombinant-subunit vaccines have offered partial protection against *T. gondii* infection (Verma and Khanna, 2013). Conversely, several attenuated vaccines, such as RHΔ*tkl1*, RHΔ*ompdc*, and ME49Δ*ldh*, have elicited a potent immune response (Fox and Bzik, 2010; Xia et al., 2018; Wang et al., 2020). These mutants cause a loss of virulence in mice because of uracil auxotrophy; in the absence of uracil *in vivo*, tachyzoites cannot propagate. With defective activity of the *de novo* pyrimidine-biosynthesis pathway, parasites fail to synthesize uridine 5′-monophosphate (UMP) for intracellular replication. These findings are extremely important for developing “ideal” vaccines against toxoplasmosis.

The genomic length of *CPSII* is 24,042 bp and it has 37 extrons, and the mRNA sequence is 5100 bp (Fox et al., 2009). The enzyme CPSII has functional domains at N- and C-terminals. The N-terminal glutamine amidotransferase domain fuses with the C-terminal carbamoyl-phosphate synthase domains, and accepts glutamine as an amine-group donor. It is an important enzyme in the *de novo* pyrimidine-biosynthesis pathway. Reports have shown that tachyzoites with CPSII disrupted, failed to replicate once intracellular without uracil supplementation (Fox and Bzik, 2002; Hortua Triana et al., 2016). The protection of *csp1-1* mutants is not always 100% and the reasons are ambiguous (Fox and Bzik, 2002, 2010, 2015). We assume that the residues of CPSII were the culprits because only part of CPSII is knocked out through homologous recombination according to early reports (Fox and Bzik, 2002; Fox et al., 2009). To achieve better understanding of the immune response induced by RHΔCPSII in mice, we generated a mutant with complete deletion of CSPII. We found that the RHΔCPSII strain reduced intracellular proliferation of tachyzoites in the absence of uracil, but the replication was not dynamic. Two to four tachyzoites in a vacuole were observed by microscopy at 48 or 72 h post-infection, which indicated the slow replication of the mutant.

To further investigate the growth patterns *in vivo*, we carried out experimental infection with RHΔCPSII strain in mice. We found that ICR mice and BALB/c mice infected with the RHΔCPSII strain lived for 10–15 days; our data are not in agreement with the report stating that the cps1-1 mutant is avirulent in mice (Fox and Bzik, 2002). We undertook PCR tests and Sanger sequencing to exclude the possibility that the RHΔCPSII strain was mixed with the wild-type strain. We assumed that *T. gondii* had evolved a compensatory pathway instead of CPSII, or the parasite had acquired a sufficient amount of uracil through the salvage pathway. In our study, no tachyzoites were found in ascitic fluid from ΔCPSII-infected mice at 5 dpi, but many parasites were found at 10 dpi, which is in accordance with our *in vitro* results. Importantly, *T. gondii* can escape the “CPSII deletion” trap, and the mechanism remains obscure. We assume that the salvage pathway takes part in the escape or the parasite evolves a bypass pathway instead of using the CPSII enzyme. The main reason why the S48 strain, as a live attenuated vaccine, is not used widely is that it may revert to gain the ability to form cysts or oocysts. A live parasite that causes no parasite burden, removes the risk of parasite transmission in the host, and elicits efficacious and long-term immune protection against further infection can be considered to be a “perfect” vaccine. Taken together, we suggest that the RHΔCPSII strain as a live vaccine needs improvement to totally eliminate the dissemination risk even though reports have declared it to be avirulent in mice (Fox and Bzik, 2002; Fox et al., 2009). An advanced strategy would be to combine multiple knockout genes from the *de novo* pyrimidine-biosynthesis pathway and salvage pathway in a single strain to completely delete uracil in designing of a live attenuated vaccine.

Despite the disadvantage mentioned above, we assessed the potential mechanism of the immune response induced by the RHΔCPSII strain for further insight. Upon infection, the percentage and the absolute number of neutrophils were increased significantly. At the early stage of infection, the parasite burden remained low in ΔCPSII group compared with WT group as shown in Figure 3, and we can infer that the immune system of mice from the ΔCPSII group was activated in order to eliminate the foreign pathogens. We also found a significant increase in the levels of IFN-γ and IL-12 in infected mice. The latter are key cytokines in immune system because they control *T. gondii* replication via cell-mediated mechanisms (Gazzinelli et al., 1994; Ivanova et al., 2019). A high level of IFN-γ can induce production of the effector proteins, immunity-related GTPases, and guanylate-binding proteins, which further damage parasitophorous vacuoles by fusion with lysosomes (Pifer and Yarovinsky, 2011). IL-12 could be produced by neutrophils and is important for IFN-γ production because it activates natural killer (NK) cells (Gazzinelli et al., 1994; Pifer and Yarovinsky, 2011). In infected mice, especially ΔCPSII-infected mice, which lived much longer, immune responses are induced by parasites and cytokines are then produced to clear the pathogens. Except for NK cells and T cells, neutrophils have been newly identified as another crucial source for the IFN-γ required for T-cell receptor-independent protection against intracellular pathogens (Sturge et al., 2013). Here, we also found notable accumulation of neutrophils after infection by the RHΔCPSII strain, which indicated that a strong immune response had been activated by this strain. The increase in neutrophils is not due to the decrease of lymphocytes by migration from blood to infected tissues, since the absolute number of neutrophils was also significantly increased in ΔCPSII-infected mice. It is not surprising that populations of cluster of differentiation (CD)4^+^ and CD8^+^ cells are significantly induced by the cps1-1 RH strain as early as 8 dpi in view of the necessity to establish immunity for controlling infection by *T. gondii* (Dupont et al., 2014). We also observed the mRNA expression of STAT1 and IRF8 to be high at 8 dpi upon infection by the RHΔCPSII strain, indicating the probability of activation of STAT1 and IRF8. STAT1 plays a part in inducing IFN-γ production, whereas IRF8 induces IL-12 expression (Majoros et al., 2017; Jefferies, 2019). Our results suggest that the RHΔCPSII strain elicits an effective cellular immune response.

## Conclusion

Our findings that a deficiency of CPSII in the RH strain leads to reduced virulence in mice suggest that vaccines targeting the *de novo* pyrimidine-biosynthesis pathway might be efficacious, and these findings could provide research basis for vaccine development. In addition, for the concern of safety and to prevent the possibility of virulence restore, we raised an advanced strategy: *T. gondii* tachyzoites that lack the *de novo* pyrimidine-biosynthesis pathway and salvage pathway, whose resources of uracil are completely shut-off, could be a much safer and promising live attenuated vaccine to prevent infection with *T. gondii*.

## Data Availability Statement

The original contributions presented in the study are included in the article/Supplementary Material. Further inquiries can be directed to the corresponding author/s.

## Ethics Statement

The animal study was reviewed and approved by the Animal Care and Use Committee of Zhejiang Academy of Medical Sciences (Zhejiang, China).

## Author Contributions

XZ, SL, HD, DL, and BZ developed the study protocol. XZ, HD, KD, and DL did the experiments. XZ analyzed the data and wrote the manuscript. SL revised the report. All authors read and approved the final manuscript.

## Conflict of Interest

The authors declare that the research was conducted in the absence of any commercial or financial relationships that could be construed as a potential conflict of interest.

## Supplementary Material

Supplementary Figure 1 Confirmation test of PCR products by sequencing. **(A)** PCR detection of CPSII5′UTR-DHFR-TS^∗^-CPSII3′UTR. **(B)** Sequences of PCR product from ΔCPSII RH strain were aligned with original sequences. Red or blue square means the sequences of 5′ or 3′ UTR of CPSII gene and the rest squares mean the sequences of DHFR gene, as shown in Figure 1A.

Supplementary Figure 2 Parasite growth *in vitro* with or without the addition of uracil. Vero cells were infected with ΔCPSII RH strain with or without the addition of 2 mM uracil. Microscope observation was carried at 24, 48, 72, and 96 hpi.
