# Supplementary material for: A Carbamoyl Phosphate Synthetase II (CPSII) Deletion Mutant of Toxoplasma gondii Induces Partial Protective Immunity in Mice
Source: Front Microbiol. 2021 Jan 14;11:616688. doi: 10.3389/fmicb.2020.616688 (PMC7840960; doi:10.3389/fmicb.2020.616688)
Supplement: Supplementary file 1 [file Table_1.DOCX]

Table 1

Primers used in this study

| primer | sequence | use |
| --- | --- | --- |
| 3'-plasmid-sgRNA | 5’-AACTTGACATCCCCATTTAC-3’ | To construct gene specific CRISPR plasmids |
| 5'-CSPII-sgRNA | 5’-GCAATGCTGGTGTTAGCCGAG  TTTTAGAGCTAGAAATAGC-3’ | To construct the *CPS II* specific CRISPR plasmid |
| 3'-CSPII-sgRNA | 5’-GAAAGAAGCGAAAGAAGCTCG  TTTTAGAGCTAGAAATAGC-3’ | To construct the *CPS II* specific CRISPR plasmid |
| gRNA2-Fw-KpnI | 5’-CGAATTGGGTACCCAAGTAAG  CAGAAGCACGCTG-3’ | To amplify the U6-sgRNA region concluding 3’-CPS II sgRNA |
| gRNA2-Rv-XhoI | 5’-TCGACCTCGAGAATTAACCCTCACTAAAGG-3’ |  |
| DHFR-csp5-F | 5’-GGATAATGGGGGTCCACCATGCGCTCGAC  GATGCGGGGGAGATGCAGAAACCGGTGTGTCT-3’ | Amplification of CPS II-DHFR* for Gibson assembly |
| DHFR-csp3-R | 5’-GAGCGAGAGATATGGAACGTTTTTGGCGAAA  GTATTGGAAACTAGACAGCCATCTCCATCT-3’ |  |
| CSPII-PCR1-F | 5’-TTGAGTCTGGCGCTACGTG-3’ | Diagnostic PCR1 |
| CSPII-PCR1-R | 5’-GCGTTGAATCTCTTGCCGAC-3’ |  |
| CSPII-PCR2-F | 5’-TTTGACGCTCATGGTTGCAC-3’ | Diagnostic PCR2 |
| CSPII-PCR2-R | 5’-AGAGGAAGAAGCATACAGCGG-3’ |  |
| CSPII-PCR3-F | 5’-CGTCTCCTCTCCGCTTCTTG-3’ | Diagnostic PCR3 |
| CSPII-PCR3-R | 5’-TACACGCGCTCATTTGCTTG-3’ |  |
| CSPII-PCR4-F | 5’-ACCAAGGGTATTCCACACGC-3’ | Diagnostic PCR4 |
| CSPII-PCR4-R | 5’-CCTTCCTCGCACTTGTGTCT-3’ |  |
| CSPII-PCR5-F | 5’-AACAGTCGACAGAGCATGGG-3’ | Diagnostic PCR5 |
| CSPII-PCR5-R | 5’-TGGAAATCCGTCGCGTAACT-3’ |  |
| CPSII-DHFR-F | 5’-GGATAATGGGGGTCCACCAT-3’ | Diagnostic PCR of CPSII5’UTR-DHFR-CPSII3’UTR |
| CPSII-DHFR-R | 5’-GAGCGAGAGATATGGAACGT-3’ |  |
| 529-bp-PCR-F | 5’-ACGAGAGTCGGAGAGGGA-3’ | Diagnostic PCR based on 529-bp |
| 529-bp-PCR-R | 5’-TGGATTCCTCTCCTACCCCT-3’ |  |
| Stat1-F | 5’-TGGGAAGTATTATTCCAGACCAAA-3’ | Amplify the stat1 gene of mice |
| Stat1-R | 5’-AGTCTTGATGTATCCAGTTCG-3’ |  |
| Irf8-F | 5’-GATCGAACAGATCGACAGCA-3’ | Amplify the irf8 gene of mice |
| Irf8-R | 5’-AAGCATCCACCTGATTG-3’ |  |
| Actin-F | 5’-GCTTCTAGGCGGACTGTTAC-3’ | Amplify the actin gene of mice |
| Actin-R | 5’-CCATGCCAATGTTGTCTCTT-3’ |  |
