# Supplementary figures and images for: A Carbamoyl Phosphate Synthetase II (CPSII) Deletion Mutant of Toxoplasma gondii Induces Partial Protective Immunity in Mice
Source: Front Microbiol. 2021 Jan 14;11:616688. doi: 10.3389/fmicb.2020.616688 (PMC7840960; doi:10.3389/fmicb.2020.616688)

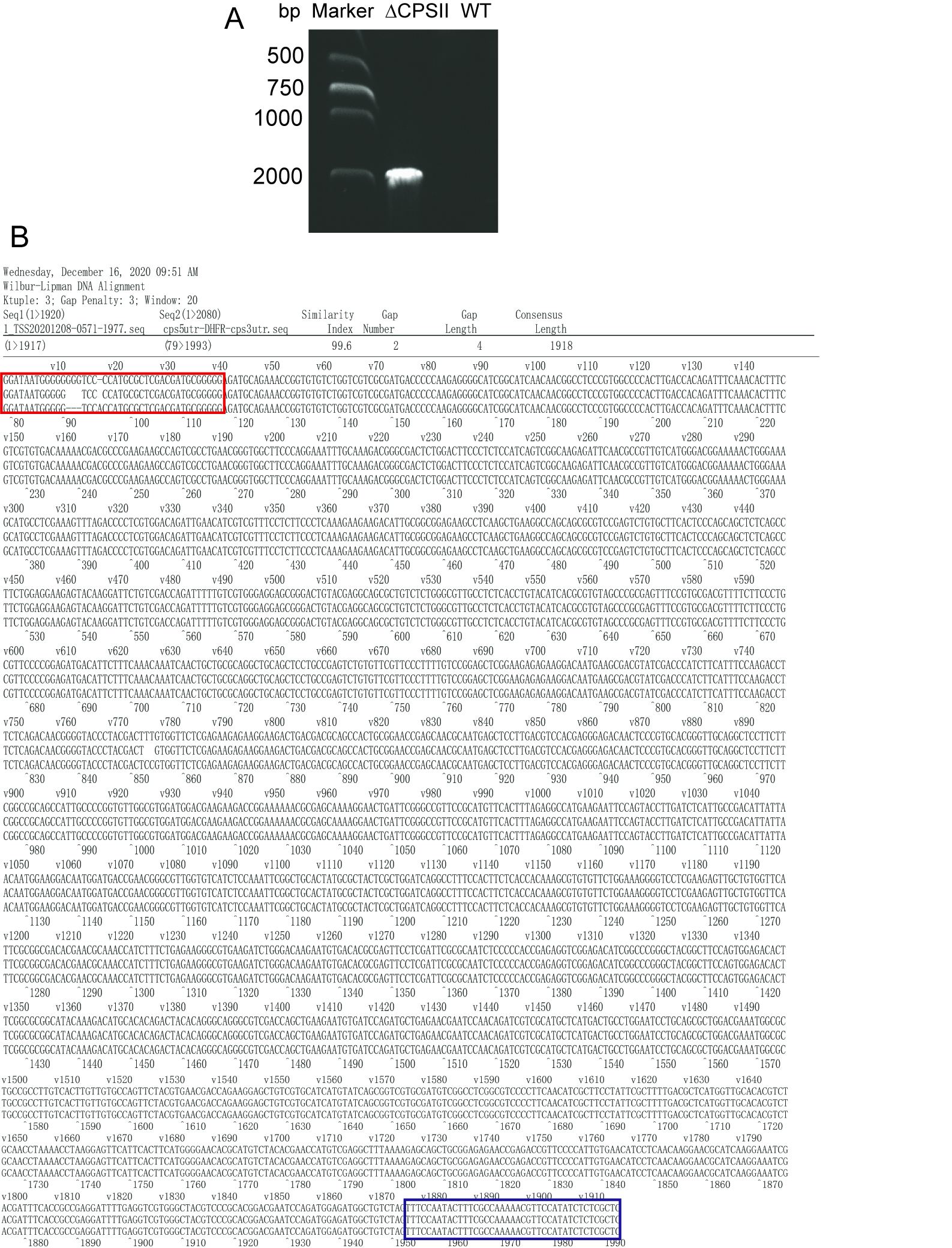

Supplement: Supplementary Figure 1 — Confirmation test of PCR products by sequencing. (A) PCR detection of CPSII5′UTR-DHFR-TS∗-CPSII3′UTR. (B) Sequences of PCR product from ΔCPSII RH strain were aligned with original sequences. Red or blue square means the sequences of 5′ or 3′ UTR of CPSII gene and the rest squares mean the sequences of DHFR gene, as shown in Figure 1A. [file Image_1.TIF]

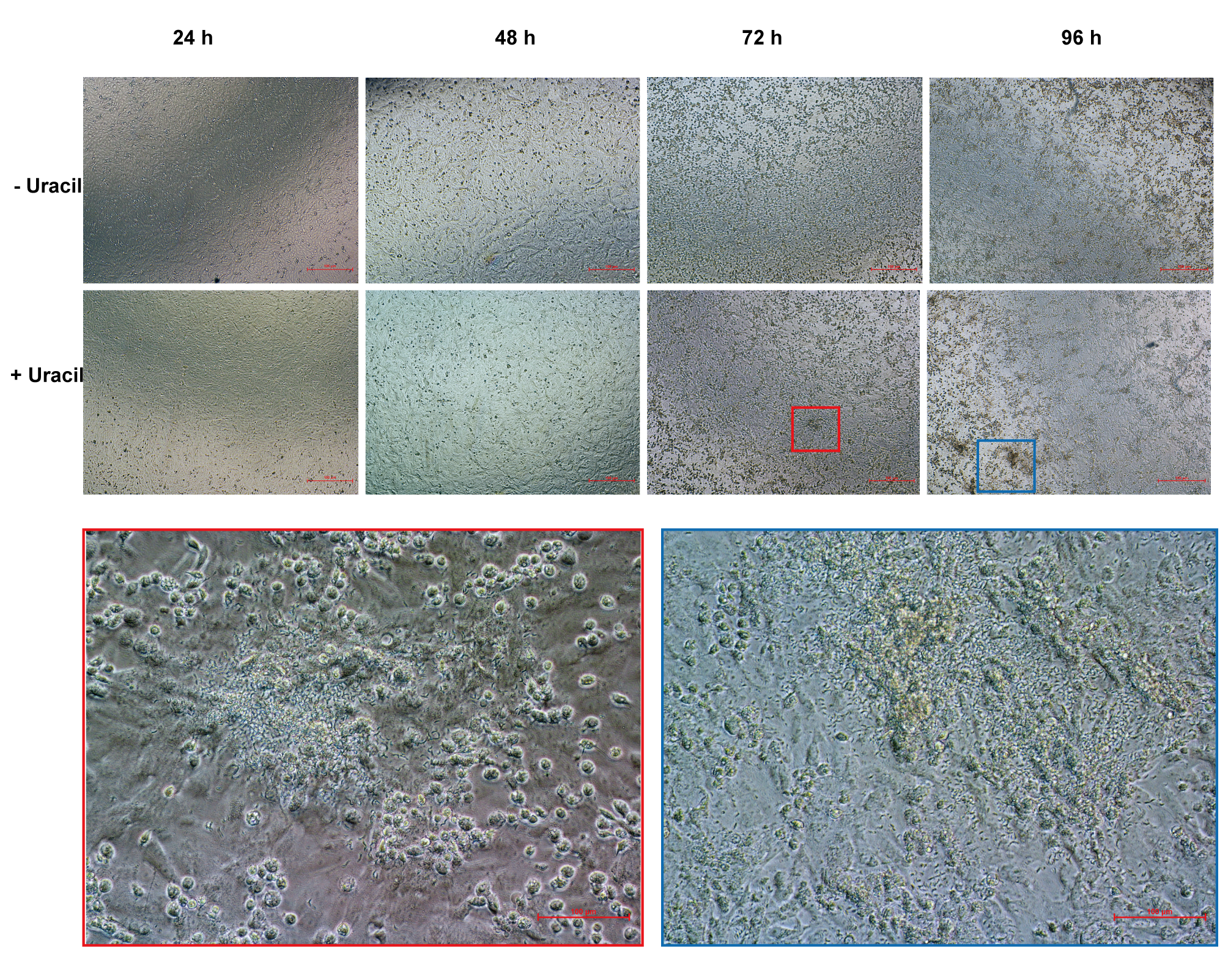

Supplement: Supplementary Figure 2 — Parasite growth in vitro with or without the addition of uracil. Vero cells were infected with ΔCPSII RH strain with or without the addition of 2 mM uracil. Microscope observation was carried at 24, 48, 72, and 96 hpi. [file Image_2.TIF]
